# Formation Flight of Multiple UAVs via Onboard Sensor Information Sharing

**DOI:** 10.3390/s150717397

**Published:** 2015-07-17

**Authors:** Chulwoo Park, Namhoon Cho, Kyunghyun Lee, Youdan Kim

**Affiliations:** Department of Mechanical & Aerospace Engineering, Seoul National University, Daehak-dong, Gwanak-gu, Seoul 151-744, Korea; E-Mails: bakgk@snu.ac.kr (C.P.); nhcho91@snu.ac.kr (N.C.); damul731@gmail.com (K.L.)

**Keywords:** multiple UAV operation, onboard sensor information sharing, monitoring multiple environment, close UAV formation, circular formation, triangular formation

## Abstract

To monitor large areas or simultaneously measure multiple points, multiple unmanned aerial vehicles (UAVs) must be flown in formation. To perform such flights, sensor information generated by each UAV should be shared via communications. Although a variety of studies have focused on the algorithms for formation flight, these studies have mainly demonstrated the performance of formation flight using numerical simulations or ground robots, which do not reflect the dynamic characteristics of UAVs. In this study, an onboard sensor information sharing system and formation flight algorithms for multiple UAVs are proposed. The communication delays of radiofrequency (RF) telemetry are analyzed to enable the implementation of the onboard sensor information sharing system. Using the sensor information sharing, the formation guidance law for multiple UAVs, which includes both a circular and close formation, is designed. The hardware system, which includes avionics and an airframe, is constructed for the proposed multi-UAV platform. A numerical simulation is performed to demonstrate the performance of the formation flight guidance and control system for multiple UAVs. Finally, a flight test is conducted to verify the proposed algorithm for the multi-UAV system.

## 1. Introduction

Many studies on multiple UAVs performing various missions have recently been conducted to address the increasing demands for unmanned aerial vehicle (UAV) applications [1,2,3,4,5,6,7,8]. Multiple UAVs can monitor multiple targets simultaneously, and multiple agents can complement each other in response to failures. A formation flight guidance law must be implemented to operate multiple UAVs. The formation flight guidance law enables each UAV to maintain relative positions in the formation, which allows the UAVs to be efficiently and safely controlled while satisfactorily performing a given mission. In [9], multiple quadrotor UAVs performed a boundary tracking formation flight while maintaining a phase angle of a geometrical boundary. In this manner, a boundary monitoring mission, such as a mission concerning an oil spill in an ocean, can be conducted rapidly and efficiently. The concept of miniature UAVs used to perform atmospheric measurements was proposed in [10]. The UAVs operated as low-cost aerial probes to measure temperature or wind profiles in the atmosphere. Therefore, multiple UAVs can effectively perform environmental monitoring missions based on formation flight guidance. The formation flight of multiple UAVs is an area wherein the robustness of the formation when subject to stochastic perturbations is crucial. Therefore, a robust analysis of the formation with respect to external disturbances, including wind, should be performed, although this is beyond the scope of this study. Additionally, the failure of a single UAV can lead to the failure of the entire system; therefore, it is important to increase the robustness of multi-UAV systems.

In the formation flight guidance law, information about other UAVs is utilized to generate formation guidance commands. Sensor information of each UAV, including position, velocity, and attitude, is measured by sensors such as instrument measurement units (IMUs), a global positioning system (GPS), and air data sensors; then, the sensor data obtained following signal processing are transferred to the other UAVs using a communication device. The sensor information of each UAV should be carefully and reliably treated and shared to successfully obtain formation flight.

Depending on the flight geometry, formation flight can be classified as a circular formation or close formation flight. Multiple UAVs typically perform a circular formation flight around an area in large-area monitoring missions. However, when the UAVs move to the next mission area, they should perform flight in close formation to reduce the total aerodynamic drag and increase survivability. Each formation guidance law has been studied and verified by numerical simulations or flight tests. A standoff tracking guidance for multiple UAVs was introduced in [11]. A nonlinear model predictive method for UAVs was proposed to track a moving target. A coordinated guidance using the second-order sliding mode was introduced in [12]. Three UAVs performed a coordinated circular formation using a standoff flight guidance law. However, the algorithms in [11,12] were developed based on the assumption that each UAV was a 3-degree-of-freedom (DOF) point-mass object. Therefore, the performance of the proposed guidance logics may be degraded when applied to a fixed-wing UAV because the longitudinal and lateral dynamics are different. A simulation environment based on a 6-DOF UAV model was introduced in [13]. The formation reconfiguration of six UAVs was performed to validate the developed environment. However, the results of the simulation may differ from the actual flight because there was an assumption of perfect information sharing between the UAVs. In contrast to the simulation environment, a communication limitation exists in the flight test. Therefore, the formation flight guidance law should be developed while considering the UAV dynamics and should be verified in the simulation while considering the communications limitations. A non-linear dynamic inversion (NLDI) guidance law was introduced in [14] to enable triangular formations. A guidance command was generated from the dynamic inversion of a simple point-mass UAV model. A simulation and a flight test of three UAVs were conducted. The cooperative formation flight of two fixed-wing UAVs was studied in [15]. A hardware-in-the-loop simulation was conducted, and a flight test that utilized the leader-follower formation pattern was performed. A cooperative controller for three rotary wing UAVs was proposed in [16] for heavy payload transport. Three engine-powered rotary UAVs formed a triangle formation, considering an unbalanced force generated by the payload. In [14,15,16], a ground control station (GCS) was used to relay information between UAVs; thus, the GCS could be a single point of formation failure. In addition, the mission range was limited within the surrounding GCS. To avoid a centralized GCS, UAVs should be interconnected using a full duplex and multipoint-to-multipoint topology to exchange information. A full duplex topology is required to enable a bidirectional connection, and a multipoint-to-multipoint topology is required for a decentralized connection between the UAVs. However, a communication system supporting this communication topology requires an expensive and heavy radiofrequency (RF) system. The system also consumes a large amount of power, which makes it unsuitable for small-scale UAV applications. Therefore, to perform a formation flight using a small UAV system, the communication system should be carefully designed by considering multiple communication topologies, low weight, low power, and low latency. This issue is why small UAVs cannot easily perform precise formation flight.

In this study, formation flight guidance algorithms are introduced and verified via both numerical simulation and a flight test using three fixed-wing UAVs. The contributions of this paper are as follows. First, an onboard sensor information-sharing system for small UAVs is developed. A wireless device is proposed considering the decentralized communication topology. Delays in the communication path are analyzed step by step, and the performance of the sensor information sharing between UAVs is verified. Second, a precise formation guidance law is developed considering the longitudinal and lateral dynamics of a fixed-wing UAV. A 6-DOF numerical simulation that includes a communication model is utilized to validate the developed guidance law. Third, an integrated formation flight test composed of various formation shapes is performed in a single flight test. Continuous circular formation flight and continuous close formation flight are conducted for a complete mission, including area monitoring and formation movement. Intermediate formation reconfiguration is also performed.

The remainder of this paper is organized as follows. In Section 2, the sensor and developed multi-UAV system are presented, as well as the system identification results. In Section 3, the onboard sensor information sharing system is explained in detail. In Section 4, a circular formation flight algorithm and close formation flight algorithm are proposed. Utilizing the algorithms, an integrated formation flight scenario is designed, and the proposed guidance algorithms are verified via a numerical simulation and flight experiment in Section 5. The conclusions of this research are presented in Section 6.

## 2. Sensor and UAV System

To perform formation flight, each UAV should be precisely controlled using sensors, and the same UAV hardware system should be used in each of the UAVs in the system to ensure uniform performance. In this study, three UAVs equipped with wind sensors were developed based on a commercial radio control (RC) aircraft. A linear dynamic model is also developed using a system identification scheme to ensure the accuracy of the numerical simulation.

### 2.1. Sensors

**Figure 1:**
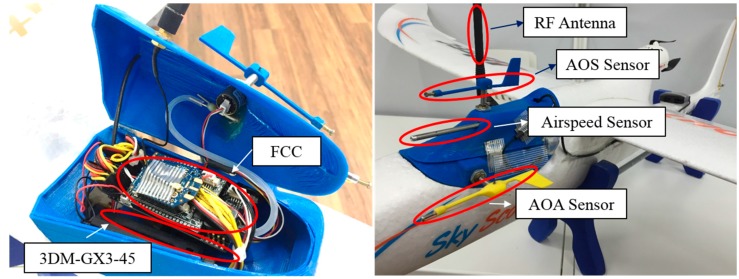
Sensors and avionics of an Unmanned Aerial Vehicle.

The developed UAV is equipped with multiple sensors to achieve precise flight control [17]. Figure 1 shows the sensors and avionics in the UAV, and detailed specifications of sensors and actuators are summarized in Table 1. A Microstrain 3DM-GX3-45 inertial navigation sensor is used to measure the vehicle attitude, position, and velocity. The external high-gain GPS antenna of the 3DM-GX3-45 is located at the tail of the UAV. A US Digital MA3 miniature absolute magnetic encoder is used to measure the angle of attack (AOA) and angle of sideslip (AOS), which are relative to the direction of the wind.

**Figure 2:**
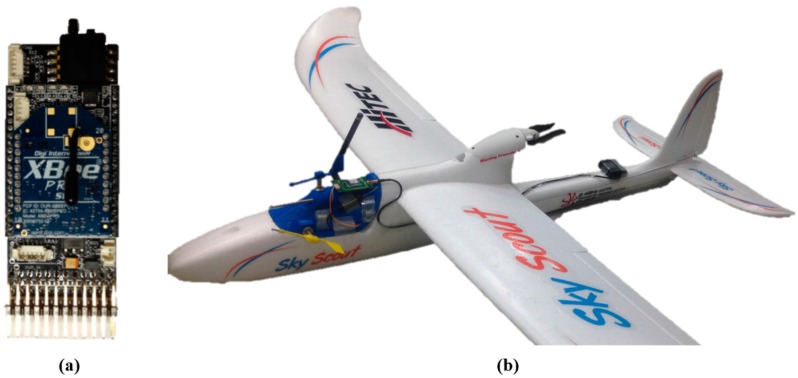
(**a**) Developed ARM Cortex-M3-based FCC; (**b**) UAV system.

This miniature encoder sensor outputs an absolute rotation angle based on a non-contact method with low friction; therefore, it is appropriate for measuring wind direction in a small-scale UAV. A pitot tube is connected to a MPX7002 differential pressure sensor to measure the relative wind speed. The measured dynamic pressure is converted to air speed by a conversion formula. All of the sensors are connected to the developed ARM Cortex-M3-based embedded flight control computer (FCC), which is shown in Figure 2a. The embedded FCC sends control commands to the control surfaces, and an onboard 900 MHz ZigBee modem communicates with the other UAVs.

**Table 1:** Sensors and avionics specifications.

Component	Model Name	Manufacturer	Data Rate	Specification
Inertial navigation sensor	3DM-GX3-45	Microstrain	50 Hz	Typ. Attitude accuracy ±0.35° Typ. Velocity accuracy ± 0.1 m/s Typ. Position accuracy ± 2.5 m RMS
AOA, AOS sensor	3MA-A10-125-B	US Digital	50 Hz	12-bit resolution, 0.08° accuracy
Airspeed sensor	MPX7002DP	Freescale	50 Hz	Typ. Pressure accuracy ± 1.6 Pa
RF telemetry	XBP09-DMUIT-156	Digi	10 Hz	3 Km LOS range, 900 MHz
FCC	ARM FCC-M3	Self-developed	400 Hz	ARM Cortex-M3, 72 MHz Clock, 6 Ch PWM In & Out

### 2.2. UAV Airframe

For reliable formation flight, identical UAVs, each with an identical avionics system, are chosen as the platform for the multi-UAV system. An off-the-shelf RC airplane is chosen to ensure uniform and reliable flight characteristics of the UAVs. The RC airplane should be selected considering the structural strength necessary to tolerate frequent takeoffs and landings as well as its portability to enable easy transportation of the UAVs. For these reasons, a pusher-type airplane (Hitec Skyscout) with a 1.4-m wingspan, as shown in Figure 2b, is selected as the UAV. A Hitec Optima 6 RC receiver and Hitec Aurora 9 RC controller are used for manual control. Using a 2200 mAh Li-Polymer battery, each UAV can fly approximately 20 min under level flight conditions.

### 2.3. UAV System Identification

An accurate dynamic model of the UAV is required to design a guidance and control algorithm for multiple UAVs. A system identification flight has been conducted to obtain a linear 6-DOF dynamic model that reflects the characteristics of the fixed-wing airplane [18,19]. During the system identification, predefined control surface inputs are used to excite the airplane dynamics, and the FCC stores the measurement data from the sensors. Lateral and longitudinal dynamic models are obtained by analyzing the recorded data. The lateral and longitudinal dynamics are identified separately. For aileron and rudder input, multistep 3-2-1-1 inputs are used to identify the lateral dynamics. For throttle and elevator control, multistep 3-2-1-1 inputs of the elevator and doublet input of the throttle are used to identify the longitudinal dynamics. The lateral and longitudinal system models are obtained as follows:

(1) $$\left[\begin{array}{c} \dot{\beta }\\ \dot{\phi }\\ \dot{p}\\ \dot{r} \end{array}\right]=\left[\begin{array}{cccc} 0 & 0.7231 & -0.1718 & -1.6319\\ 0 & 0 & 0.9957 & -0.0942\\ -53.0053 & 0 & -10.5207 & 10.4250\\ 7.3627 & 0 & -3.3925 & -4.8898 \end{array}\right]\left[\begin{array}{c} \beta \\ \phi \\ p\\ r \end{array}\right]+\left[\begin{array}{c} \begin{array}{cc} -1.4129 & -1.7054 \end{array}\\ \begin{array}{cc} 0 & 0 \end{array}\\ \begin{array}{cc} -51.1925 & 8.2723 \end{array}\\ \begin{array}{cc} -9.0546 & -13.6097 \end{array} \end{array}\right]\left[\begin{array}{c} \delta_{ail}\\ \delta_{rud} \end{array}\right]$$

(2) $$\left[\begin{array}{c} \dot{V}\\ \dot{\alpha }\\ \dot{\theta }\\ \dot{q} \end{array}\right]=\left[\begin{array}{cccc} 0 & 25.0414 & -9.7545 & 0\\ -0.4130 & -12.0086 & 0.9435 & 2.2203\\ 0 & 0 & 0 & 1\\ -0.1204 & -23.5155 & 0 & -0.6636 \end{array}\right]\left[\begin{array}{c} V\\ \alpha \\ \theta \\ q \end{array}\right]+\left[\begin{array}{c} \begin{array}{cc} 0.0080 & 10.0711 \end{array}\\ \begin{array}{cc} 0.0003 & 3.2113 \end{array}\\ \begin{array}{cc} 0 & 0 \end{array}\\ \begin{array}{cc} -0.0039 & -15.3344 \end{array} \end{array}\right]\left[\begin{array}{c} \delta_{thr}\\ \delta_{ele} \end{array}\right]$$

**Figure 3:**
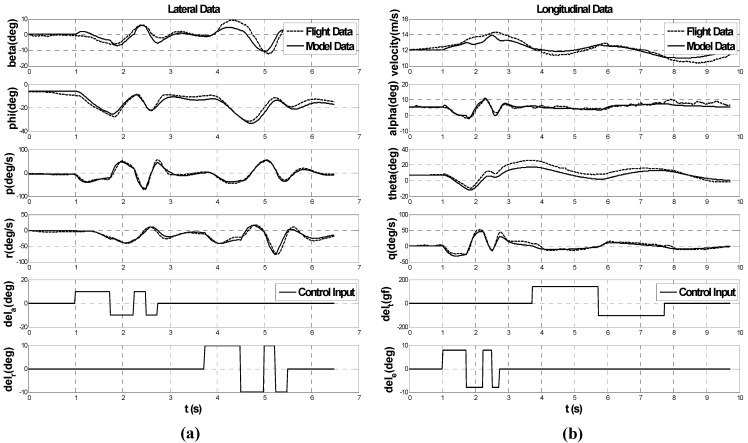
(**a**) Lateral; and (**b**) longitudinal system ID results for target airplane.

Figure 3 shows the lateral and longitudinal system identification results with the corresponding control inputs. System responses of the acquired model (solid line in Figure 3) are well matched with the recorded flight results (dashed line in Figure 3). The estimated lateral and longitudinal dynamic models are implemented in a MATLAB/Simulink environment to perform the 6-DOF simulation.

## 3. Onboard Sensor Information Sharing via Wireless Communication

Developing a method of sensor information sharing is a challenging issue in multi-UAV operation because unsynchronized information may cause incorrect decisions to be made when incorrect sensor information is shared among the UAVs. If all UAV communication is relayed via a leader aircraft or ground control system, then the central system may become a single point of failure, which would result in the UAVs having a limited mission range. Therefore, decentralized communication should be realized. In the actual flight environment, communication is conducted via a wireless device; as a result, the device should be selected considering both synchronization and decentralization.

Typical wireless communication devices are summarized in Table 2. In general, Wi-Fi is widely used for wireless communication. However, Wi-Fi requires a router to connect individual devices; therefore, decentralized communication cannot be realized. Bluetooth has a relatively short communication range and only provides a connection to a single device. In contrast, the ZigBee modem can communicate with multiple devices without a router and can cover a large distance. Therefore, the ZigBee modem (Digi XBP09) is selected as the wireless device for the system. In particular, the ZigBee modem acts as a transparent transceiver without requiring additional encoding/decoding, thereby enabling real-time communication.

**Table 2:** Comparison of wireless technology standards.

	Wi-Fi	Bluetooth	ZigBee
**Range**	50–100 m	10–100 m	100 m–1 km
**Network Topology**	Point to Hub Ad-hoc	Ad-hoc	Ad-hoc, peer to peer, star, mesh
**Frequency**	2.4 GHz and 5 GHz	2.4 GHz	868 MHz, 900 MHz, 2.4 GHz
**Complexity**	High	High	Low
**Power Consumption**	High	Middle	Very low
**Security**	WEP	64-bit or 128-bit encryption	128 AES or AL
**Applications**	-Wireless LAN	-Wireless device connection	-Industrial monitoring-Sensor network

Using the selected wireless device, *i.e*., the ZigBee modem, UAVs share their sensor information with each other. The sensor information to be shared includes the UAV’s status, such as attitude, position, velocity, and other essential parameters. Table 3 presents the communication packet used in the developed multi-UAV system. In Table 1, Vel denotes a velocity, Air denotes a barometric output, and Cmd denotes a command generated from the guidance algorithm.

**Table 3:** Communication packets of the multi-UAV system.

Data	Header	Status	Latitude	Longitude	Height	GPS Time	Roll	Pitch	Yaw	P
Byte	1–2	3–6	7–10	11–14	15–18	19–22	23–26	27–30	31–34	35–38
Q	R	Vel N	Vel E	Vel D	Acc X	Acc Y	Acc Z	Air Height	Air speed	Gamma Cmd
39–42	43–46	47–50	51–54	55–58	59–62	63–66	67–70	71–74	75–78	79–82
Roll Cmd	Battery	Elevator	Rudder	Throttle	Aileron	Alpha	Beta	Stage	Reserved	Putter
83–86	87–90	91–94	95–98	99–102	103–106	107–110	111–114	115–118	119–122	12–124

**Figure 4:**
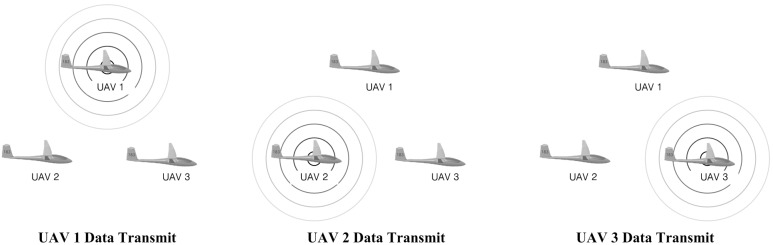
Description of sequential cyclic communication.

**Figure 5:**
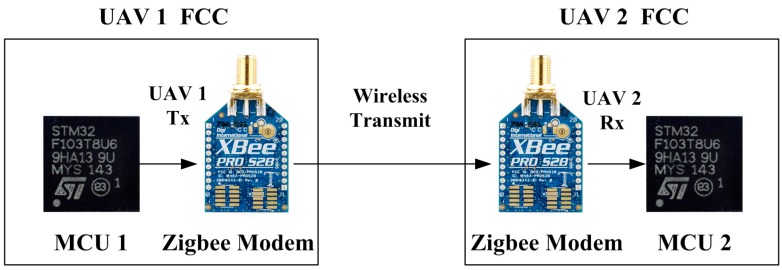
Communication flow for multiple UAVs.

To achieve robust onboard sensor information sharing between UAVs, a sequential cyclic communication method is used, as shown in Figure 4. The trigger UAV (UAV1) starts broadcasting its sensor information. Next, the neighboring UAV (UAV2) transmits its sensor information. This procedure continues until the last UAV (UAV3) performs a transmission. This procedure can prevent data loss from occurring due to simultaneous data transmission. The concept of sequential cyclic communication is realized at a low information sharing rate (<1 Hz). However, this approach requires accurate timing control to achieve a high information sharing rate (>10 Hz) because physical delays may exist during data transmission. Figure 5 describes a communication flow in the FCC. Three major physical delays can be found in the communication flow. The first delay is from microprocessor 1 (MCU1) to Zigbee1, $t_{MCU1toZigbee1}$. If UAV1 data consist of *n* bytes (8*n* bits) and the baud rate is *P* bps (bits per second), then the delay from MCU1 to Zigbee1 can be calculated as follows:

(3) $$t_{MCU1toZigbee1}=\frac{8n}{p}$$

The second delay is from Zigbee1 to Zigbee2, $t_{Zigbee1\;to\;Zigbee2}$. This delay depends on the air rate specification, that is, the RF transmission speed in air, which is inversely proportional to the carrier frequency of the modem. The data processing time inside the ZigBee modem is also included in that delay.

If the ZigBee modem has a baud rate of *P* bps, then the delay from Zigbee1 to Zigbee2 can be calculated as:

(4) $$t_{Zigbee1\;to\;Zigbee2}=\frac{8n}{q}+t_{Zigbee\;processing}$$

The third delay is from Zigbee2 to MCU2, $t_{Zigbee2toMCU2}$, which is equal to $t_{MCU1toZigbee1}$.

(5) $$t_{Zigbee\;2\;to\;MCU2}=\frac{8n}{p}$$

Therefore, the total communication delay can be expressed as:

(6) $$t_{total}=t_{MCU1toZigbee1}+t_{Zigbee1\;to\;Zigbee2}+t_{Zigbee2toMCU2}$$

**Figure 6:**
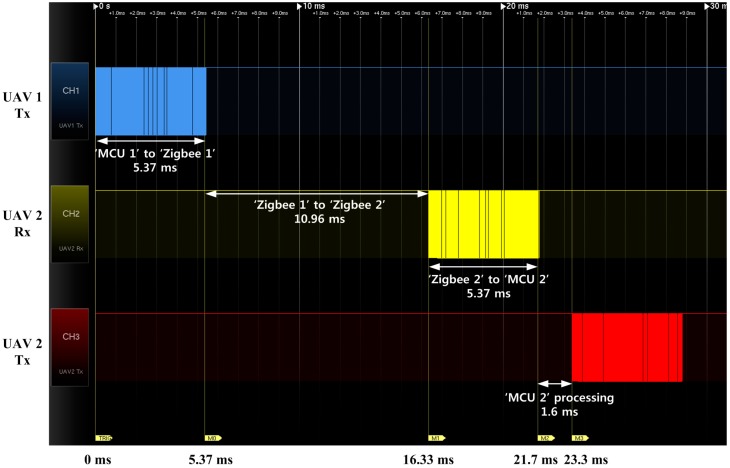
ZigBee communication delay between UAV1 and UAV2.

**Figure 7:**
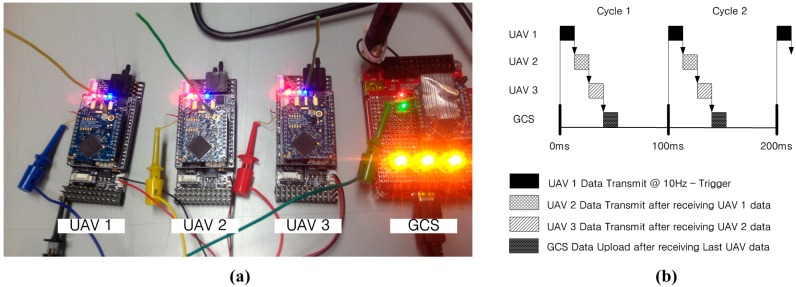
(**a**) Embedded FCCs and GCS; (**b**) sequential cyclic communication.

The considered ZigBee modem, Xbee-Pro DigiMesh900, has a baud rate of 230,400 bps and an air rate of 156,000 bps. In this case, nearly 24 ms passes between the first transmission to the second transmission for the *n* = 124-byte case. The total communication delay can be measured using a signal analyzer; as shown in Figure 6, 10 Hz of onboard sensor information sharing is possible because one cycle of four UAVs takes 24 ms × 4 = 96 ms. Accurate 10 Hz cyclic communication can be achieved if the FCC has less than 4 ms of additional processing delay. Because the typical minimum time interval of Microsoft Windows OS is in the range of 10 ms–20 ms, the Windows OS is not suitable for handling the cyclic communication. In this study, a real-time embedded FCC is used because it can control the communication timing with a 1 ms resolution. The developed embedded FCC and cyclic communication sequence is shown in Figure 7.

**Figure 8:**
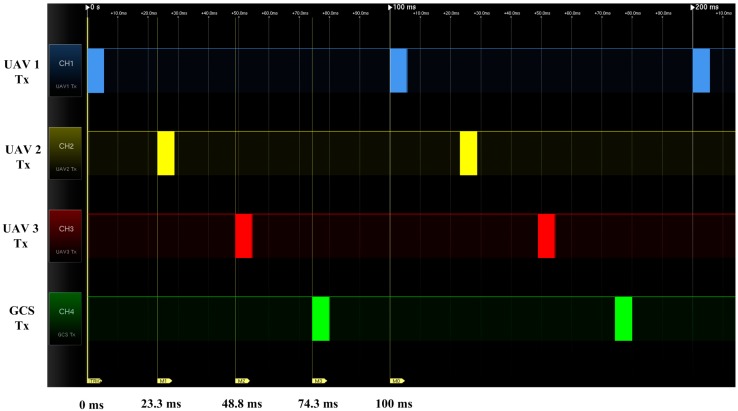
Timing measurement result of sequential cyclic communication.

When UAV1 transmits its own data to other UAVs using the ZigBee modem, other UAVs receive the data. A periodic 1 ms watching process checks the received data, and the next UAV transmits its own data according to the given transmission order. The GCS also acts as a virtual UAV for monitoring and command uploading purposes, which does not affect the formation flight or communication structure. To verify the performance of the sequential cyclic communication, the transmission timing of the FCCs is measured, as shown in Figure 8. The sequential cyclic communication is found to function well, with 10 Hz onboard sensor information sharing properly conducted among the UAVs.

## 4. Guidance Algorithms for Formation Flight

The formation flight of multiple UAVs requires various formation flight algorithms for large-area monitoring and formation movement. Circular formation flight guidance is suitable for the omni-directional monitoring of large areas because such guidance can control the phase angles among UAVs on the circular path. However, close formation flight guidance is proper for the polygonal shape formation of fixed-wing UAVs because such guidance can decrease the aerodynamic drag when flying to the next mission area. These algorithms are explained in Section 4.1 and Section 4.2, respectively.

### 4.1. Circular Formation Flight Guidance

**Figure 9:**
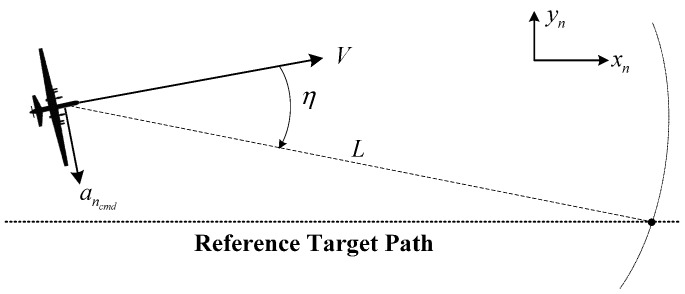
Nonlinear path-following guidance.

When a fixed-wing UAV monitors an area, a loitering flight is typically performed. If multiple UAVs are monitoring the area, the phase angles between UAVs should be controlled to ensure efficient area surveillance on the circular path. Nonlinear path-following guidance [20,21,22] was proposed to make UAVs fly on a circular path, assuming that all UAVs are moving on a two-dimensional surface, *i.e*., flying at the same altitude. The stability of nonlinear path-following guidance was proven using the Lyapunov stability theorem [20], and therefore, all UAVs asymptotically converge to the predefined path. The reference altitude is set sufficiently high to cover the ground slopes and hills. The lateral guidance geometry of the nonlinear path following is shown in Figure 9, where *V* is the airspeed, *L* is the constant guidance distance, and η is the angle between *V* and *L.*

The UAV can be guided to the reference path by the following lateral acceleration command $a_{n_{cmd}}$:

(7) $$a_{n_{cmd}}=\frac{2V^{2}}{L}\sin\eta$$

The following roll command can be used to make the UAV follow the lateral acceleration command $a_{n_{cmd}}$:

(8) $$\phi_{cmd}=\tan^{-1}\left(\frac{a_{n_{cmd}}}{g}\right)$$

where *g* is the acceleration of gravity. Wind and sideslip motion are not considered in this guidance law.

**Figure 10:**
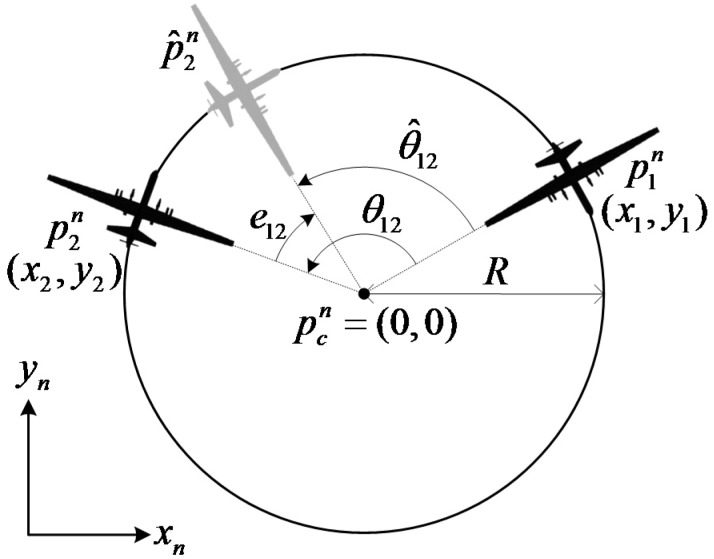
Phase angle on a circular path.

A circular path can be generated from the target point $p_{c}^{n}$ and the loitering radius *R*, as shown in Figure 10. The target point $p_{c}^{n}$ is assumed to be the origin (0,0) without loss of generality. Once the UAVs are flying on the circular path using the nonlinear path following guidance, a phase angle can be maintained by controlling the airspeed of the UAV.

In this study, the relative phase angles of three UAVs are calculated in counter-clockwise order, starting from the first UAV. The phase angle between the UAVs is calculated using the positions $p_{1}^{n}$ and $p_{2}^{n}$ of the UAVs as:

(9) $$\theta_{12}=atan2(y_{2}-y_{1},x_{2}-x_{1})$$

Because the phase angle starts from the 1st UAV, it has a positive value in the range of [0,2π). The target phase angle ${\hat{\theta }}_{12}$ is determined by considering the number of UAVs. Using the phase angle $\theta_{12}$ and target phase angle ${\hat{\theta }}_{12}$, the phase angle error $e_{12}$ can be calculated as:

(10) $$e_{12}={\hat{\theta }}_{12}-\theta_{12}$$

A speed command for controlling the phase angle can be generated using the phase angle error as:

(11) $$V_{cmd}=V_{cruise}+sat(K_{v}e_{12}),K_{v}>0$$

where $V_{cruise}$ is the cruise velocity during level flight and $K_{v}$ is a proportional velocity guidance gain. To prevent each UAV from entering a stall speed, a saturation value ΔV is set to $K_{v}e_{12}$ in Equation (11). In addition, the flight path angle command, $\gamma_{cmd}$, is generated to track the target height $h_{ref}$ as follows:

(12) $$\begin{array}{l} h_{err}=h_{ref}-h\\ \gamma_{cmd}=K_{p}h_{err}+K_{i}\int h_{err}dt+K_{d}{\dot{h}}_{err} \end{array}$$

where $K_{p},K_{i},K_{d}$ are the proportional, integral, and derivative gains of the height controller, respectively.

### 4.2. Close Formation Flight Guidance

**Figure 11:**
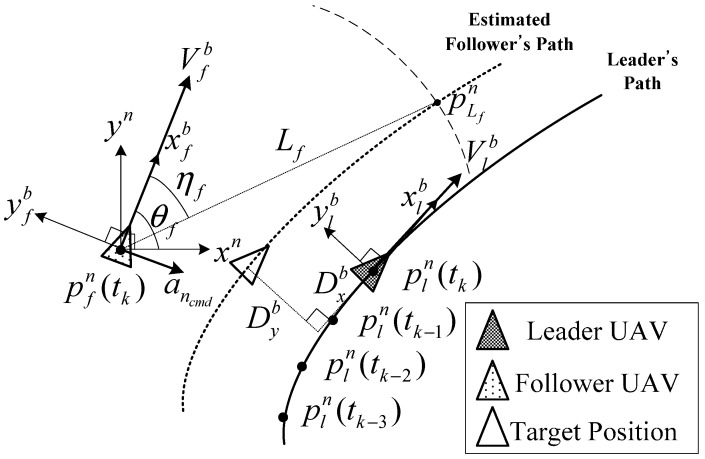
Formation path generation of a follower UAV.

For close formation flight of the fixed-wing UAVs, the use of the separated design of the longitudinal and lateral guidance laws increases the performance of the formation flight because fixed-wing airplanes have different flight characteristics in the longitudinal and lateral axes. The lateral guidance law of the close formation flight is designed based on the nonlinear path-following guidance, as shown in Figure 11. In this study, the UAVs in the close formation are classified as a leader or as followers. The leader UAV follows the prescribed target path, and it shares all of the sensor information with the followers. The follower UAVs estimate the leader’s path based on the most recent position data of the leader UAV. Using the estimated path of the leader UAV, the follower UAVs generate their own formation paths using the formation guidance law. Figure 11 shows a geometric description of the path estimation process.

In Figure 11, $\left(x^{n},y^{n}\right)$ denotes an inertial navigation frame, $\left(x_{l}^{b},y_{l}^{b}\right)$ denotes the body frame of the leader UAV, $\left(x_{f}^{b},y_{f}^{b}\right)$ denotes the body-fixed frame of the follower UAV, and $\left(D_{x}^{b},D_{y}^{b}\right)$ is the distance of the desired longitudinal and lateral formation between the leader and follower. The subsequent position data of the leader UAV, $p_{l}^{n}(t_{k})$, are recorded by the follower UAV to estimate the leader’s future trajectory.

(13) $$p_{l}^{n}(t_{k})=\left[\begin{array}{c} x_{l}^{n}(t_{k})\\ y_{l}^{n}(t_{k}) \end{array}\right],t_{k}=t_{0}+kT,k=0,1,2,3$$

The recoded positions depend on a communication interval T. To estimate the leader’s path based on the follower’s position, the follower’s position $p_{f}^{n}$ is subtracted from $p_{l}^{n}(t_{k})$ as

(14) $$\Delta p_{f}^{n}=p_{l}^{n}-p_{f}^{n},\{\begin{array}{c} \Delta p^{n}(t_{k})=p_{l}^{n}(t_{k})-p_{f}^{n}(t_{k})\\ \Delta p^{n}(t_{k-1})=p_{l}^{n}(t_{k-1})-p_{f}^{n}(t_{k})\\ \Delta p^{n}(t_{k-2})=p_{l}^{n}(t_{k-2})-p_{f}^{n}(t_{k})\\ \Delta p^{n}(t_{k-3})=p_{l}^{n}(t_{k-3})-p_{f}^{n}(t_{k}) \end{array}$$

The relative position $\Delta p_{f}^{n}$ is transformed into the follower’s body frame axis $x_{f}^{b}$ using the follower’s heading angle $\theta_{f}$ as:

(15) $$\Delta p_{f}^{b}=\Omega_{n}^{b}\times \Delta p_{f}^{n},\Omega_{n}^{b}=\left[\begin{array}{cc} \cos(\theta_{f}) & -\sin(\theta_{f})\\ \sin(\theta_{f}) & \cos(\theta_{f}) \end{array}\right]$$

Using the relative position $\Delta p_{f}^{b}(t_{k})={\left[\Delta x_{f}^{b}(t_{k})\Delta y_{f}^{b}(t_{k})\right]}^{T}$, the coefficients of the cubic polynomial function ${\left[c_{11}c_{21}c_{31}c_{41}\right]}^{T}$ can be calculated using the least squares method.

(16) $$\left[\begin{array}{c} c_{11}\\ c_{21}\\ c_{31}\\ c_{41} \end{array}\right]={\left(A^{T}A\right)}^{-1}A^{T}Y,A=\left[\begin{array}{cccc} {\left(\Delta x_{f}^{b}(t_{k})\right)}^{3} & {\left(\Delta x_{f}^{b}(t_{k})\right)}^{2} & \Delta x_{f}^{b}(t_{k}) & 1\\ {\left(\Delta x_{f}^{b}(t_{k-1})\right)}^{3} & {\left(\Delta x_{f}^{b}(t_{k-1})\right)}^{2} & \Delta x_{f}^{b}(t_{k-1}) & 1\\ {\left(\Delta x_{f}^{b}(t_{k-2})\right)}^{3} & {\left(\Delta x_{f}^{b}(t_{k-2})\right)}^{2} & \Delta x_{f}^{b}(t_{k-2}) & 1\\ {\left(\Delta x_{f}^{b}(t_{k-3})\right)}^{3} & {\left(\Delta x_{f}^{b}(t_{k-3})\right)}^{2} & \Delta x_{f}^{b}(t_{k-3}) & 1 \end{array}\right],Y=\left[\begin{array}{c} \Delta y_{f}^{b}(t_{k})\\ \Delta y_{f}^{b}(t_{k-1})\\ \Delta y_{f}^{b}(t_{k-2})\\ \Delta y_{f}^{b}(t_{k-3}) \end{array}\right]$$

The polynomial path function of the leader UAV can be estimated as:

(17) $${\hat{l}}_{l}^{b}(x)=c_{11}{\left(x_{f}^{b}\right)}^{3}+c_{21}{\left(x_{f}^{b}\right)}^{2}+c_{31}x_{f}^{b}+c_{41}$$

Based on the estimated polynomial path function ${\hat{l}}_{l}^{b}(x)$, the follower UAV generates a formation path $l_{f}^{b}(x)$ with the lateral distance $D_{y}^{b}$ aligned with the $y_{f}^{b}$-axis as

(18) $$l_{f}^{b}(x)={\hat{l}}_{l}^{b}(x)+D_{y}^{b}$$

The follower UAV can follow the generated formation path by applying the nonlinear path-following guidance law. A roll angle $\phi_{cmd}$ command is calculated using the lateral acceleration command $a_{n_{cmd}}$ as

(19) ϕcmd=tan−1(ancmdg)where ancmd=2Vf2LLfsinηf

Finally, the attitude controller makes the UAV follow the roll angle command $\phi_{cmd}$.

Once The follower UAV is on the formation path, a longitudinal velocity command $V_{ref}$ is generated to control the longitudinal distance $D_{x}^{b}$ aligned to the $x_{f}^{b}$-axis as

(20) $$V_{cmd}=V_{cruise}-K_{d}\left(D_{x}^{b}-(x_{l}^{b}-x_{f}^{b})\right)$$

## 5. Simulation and Experimental Results

### 5.1. Procedure for Autonomous Formation Flight

A formation flight scenario that consists of area monitoring and formation movement is conducted to demonstrate the performance of the formation flight guidance laws. In the area monitoring scenario, the UAVs circle the area based on the circular formation flight guidance. In the formation movement scenario, the UAVs fly closely in a triangular formation as they move to the next target point area.

**Figure 12:**
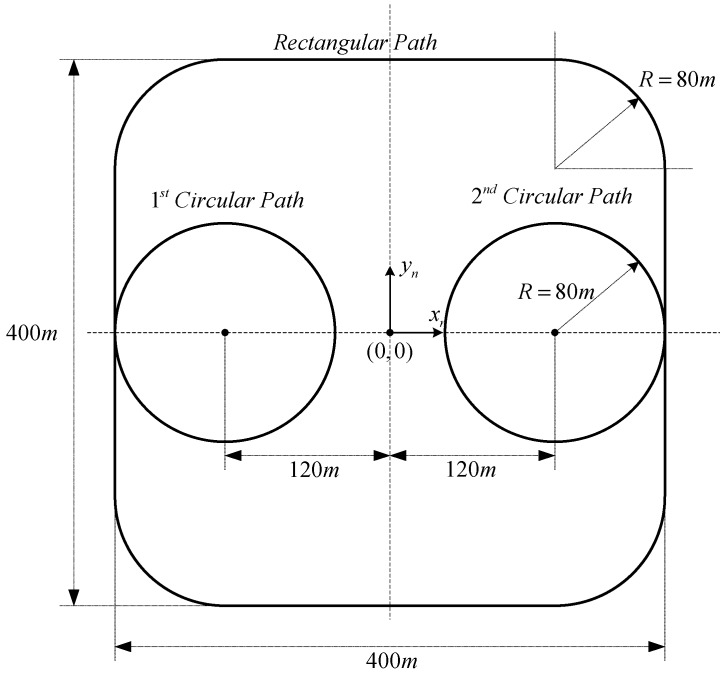
Path description of the integrated formation flight scenarios.

An integrated formation flight is introduced with five subscenarios, which are conducted in sequence. Table 4 summarizes the formation flight scenarios considered in this study. Each scenario has a specified mission and stage number depending on the longitudinal and lateral guidance mode. The mission variable specifies the observation or movement scenario, and the stage variable addresses the guidance mode transition. During the integrated formation flight, the stage variable is automatically changed based on the consensus of the UAVs. Figure 12 shows the path description of the integrated formation flight scenarios. Five subscenarios are described below.

**Table 4:** Integrated formation flight scenarios.

Scenario	Maneuver	Mission	Stage	Longitudinal Guidance	Lateral Guidance
-	Sequential takeoff	-	-	-	-
1	Circular formation/Separated altitude	1	1,2	$360/N_{1}\deg$ phase separation/δh=±10 m	Circular path following approximately 1st target
2	Circular formation/ Same altitude	1	3	$360/N_{1}\deg$ phase separation/δh=0 m	Circular path following approximately 1st target
3	Separation and Reconfiguration ofCircular formation	1	4,5	$360/N_{2}\deg$ phase separation/δh=0 m	Transition from 1st target to 2nd target
4	Close circular formation	1	6	30deg phase separation/δh=0 m	Circular path following approximately 2nd target
5	Close triangular formation	2	-	−10 m rear position of leader UAV/δh=0 m	±10 m left/right position of leader UAV
-	Sequential landing	-	-	Longitudinal guidance	-

#### 5.1.1. Circular Formation Flight with Separated Altitude

In the flight experiment, takeoff and landing are manually conducted by human pilots. Three UAVs takeoff in sequence, and the manual mode of each UAV is immediately switched to autonomous formation flight mode when it reaches the reference height. To prevent collisions between the UAVs during takeoff, each UAV follows a circular path with an altitude difference δh. The reference altitude is UAV01 = 60 m, and the altitude difference between UAV2 and UAV3 is set as δh=±10 m; therefore, UAV2 is at a target altitude of 50 m, and UAV3 is at 70 m (Stage 1). During the separated altitude flight, the relative phase angles among UAVs are regulated to $360/N_{1}\deg$, where $N_{1}$ is a number of UAVs. The relative phase angles are controlled by the circular formation flight guidance law. If all three UAVs are on the circular path with 120±10 deg phase angles (Stage 2), then UAV2 and UAV3 move to the reference altitude 60 m by increasing/decreasing their altitude via a flight path angle control.

#### 5.1.2. Circular Formation Flight at the Same Altitude

During the altitude transitions, the phase angles among the UAVs may change because of the acceleration of UAV3 and the deceleration of UAV2. Phase angle formation flight guidance regulates the phase angles of the UAVs to be 120 ± 10 deg (Stage 3). In this scenario, omni-directional surveillance of the target can be performed, wherein the target is at the center of the circular path.

#### 5.1.3. Separation and Reconfiguration Formation Flight of the Circular Formation

To monitor multiple areas, the UAVs on the circular path should be separated and move to the second target. In the separation stage (Stage 4), the UAVs fly from the 1st circular path to the 2nd circular path one by one. During the separation, the phase angles of the remaining UAVs are modified to the phase angle of $360/N_{1}\deg$, where $N_{1}$ is the number of UAVs remaining. In the reconfiguration stage (Stage 5), UAVs on the 2nd circular path are reconfigured to maintain a phase angle of $360/N_{2}\deg$, where $N_{2}$ is the number of the UAVs in the 2nd circular path. In this stage, UAVs can monitor multiple areas while the circular formation flight is performed.

#### 5.1.4. Close Circular Formation Flight

After the UAVs complete their monitoring of the mission area, they should fly together to the next mission area while maintaining the close formation flight. To perform this task, the UAVs on the circular path should converge to establish a close formation. To accomplish this maneuver, the phase angles between the UAVs are adjusted to 30 ± 10 deg in this stage (Stage 6). Once the specified phase angle has been reached, the circular formation flight mode is switched to the close formation flight mode.

#### 5.1.5. Close Triangular Formation Flight

The close formation flight guidance law makes three UAVs follow a prescribed path while keeping them in a triangular formation. In the triangular configuration, UAV1, UAV2, and UAV3 are located at (0 m,0 m) (−10 m,−10 m), and (−10 m,10 m), respectively. UAV1 becomes the leader UAV, which tracks the predefined path, and the other UAVs become follower UAVs. The formation path of each of the followers can be calculated based on the leader’s path. To ensure collision avoidance, a safety radius of 2 m is considered for each of the following UAVs. If one of the follower UAV’s relative distance becomes less than 2 m, then an additional lateral command is activated to make the follower UAV fly at a distance from the formation.

### 5.2. Simulation Results

**Figure 13:**
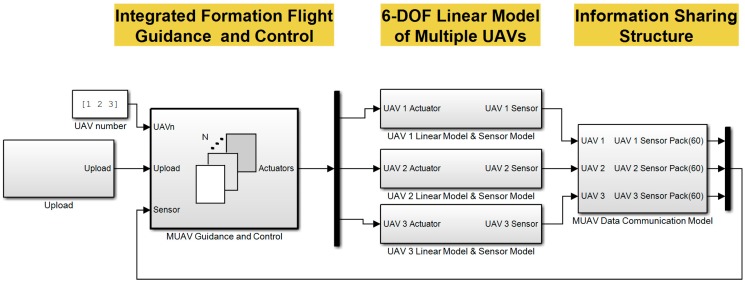
Simulation configuration in MATLAB/Simulink.

The proposed integrated formation flight scenario is composed of switching logics and multiple formation flights. The algorithms are thoroughly examined using a 6-DOF numerical simulation in the MATLAB/Simulink environment. The onboard sensor information sharing method and communication delay are considered in the simulation to emulate a real multi-UAV environment. The simulation blocks of the MATLAB/Simulink are shown in Figure 13. The identical integrated formation flight guidance block is used in all UAVs with the assigned UAV number. The sensor information sharing block emulates data transfer among the UAVs. Different execution rates of the guidance loop and communication loop are also implemented in the simulation block.

**Figure 14:**
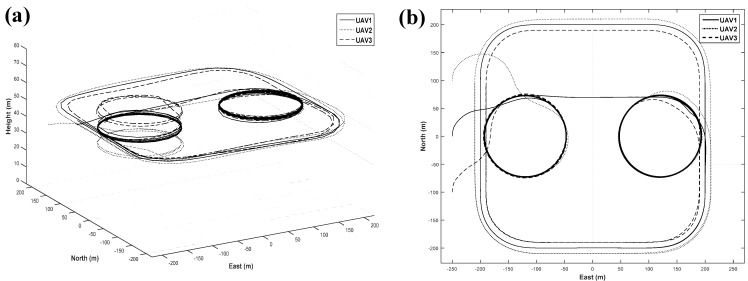
(**a**) 3D; and (**b**) 2D simulation results of integrated formation flight.

The numerical simulation results are shown in Figure 14 and indicate that the formation flight of multiple UAVs is performed well using both the close formation flight algorithm and close formation flight algorithm.

### 5.3. Experimental Results

After demonstrating the performance of the proposed algorithm via numerical simulation, a flight test is performed using the developed multi-UAV system. The integrated formation guidance algorithm block is directly converted into embedded C code via the MATLAB/Embedded Coder^®^.

**Figure 15:**
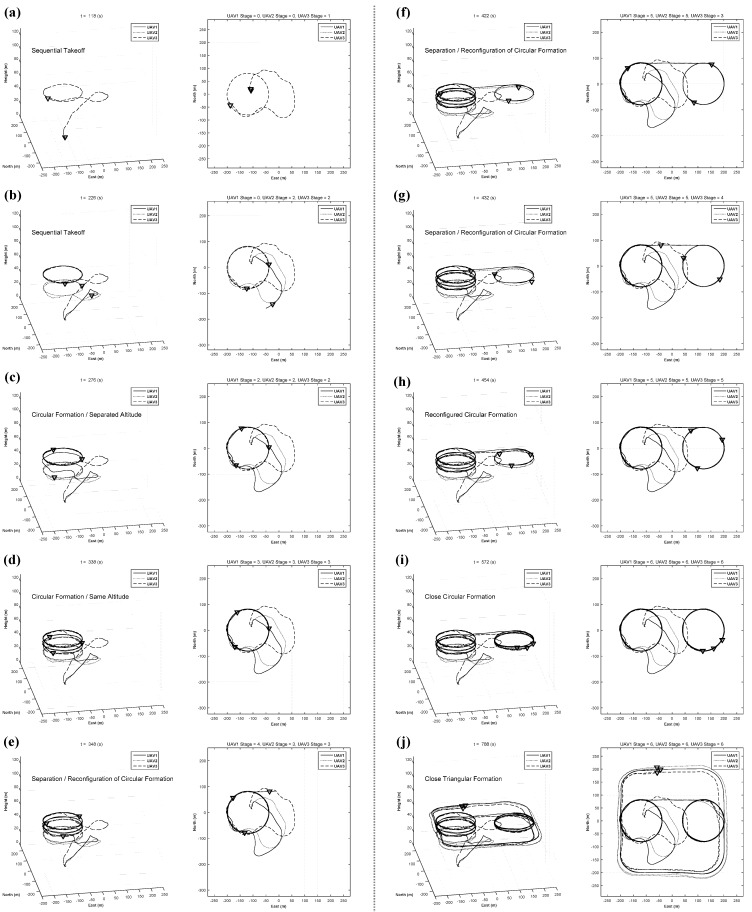
Flight results of integrated formation flight: (**a**)–(**b**) sequential takeoff; (**c**) circular formation at different altitudes; (**d**) circular formation at same altitude; (**e**)–(**h**) separation and reconfiguration of circular formation; (**i**) close circular formation; (**j**) close triangular formation.

The integrated formation flight scenario in Table 4 is conducted in sequence. The Mission and State variables are sequentially and automatically changed. The fight test is conducted in an area that measures 400 m × 400 m, and the results are shown in Figure 15. The integrated formation flight was conducted successfully. In Figure 15, solid, dotted, and dashed lines correspond to the trajectories of UAV1, UAV2, and UAV3, respectively. During the sequential takeoff stage (Figure 15a,b), the UAVs form a 120-degree circular formation at different altitudes to ensure safety (Figure 15c). Once the phase angle of the circular formation is stabilized, UAV2 and UAV3 move to the same altitude, 60 m, and execute circular formation flight (Figure 15d). After performing a circular formation flight along the circular path, UAV1 is separated from the formation and moves to the next circular path, and UAV2 and UAV3 continue the circular formation of 180° along the first circular path (Figure 15e). UAV2 moves to the second circular path and reconfigures the 180-degree circular formation with UAV1 (Figure 15f) at the second circular path. Next, UAV3 moves to the second circular path (Figure 15g), and finally, the UAVs reconfigure the circular formation of 120° (Figure 15h). The UAVs reduce the phase angles to 30° to prepare for close formation flight (Figure 15i); then, they perform the close triangular formation flight (Figure 15j).

**Figure 16:**
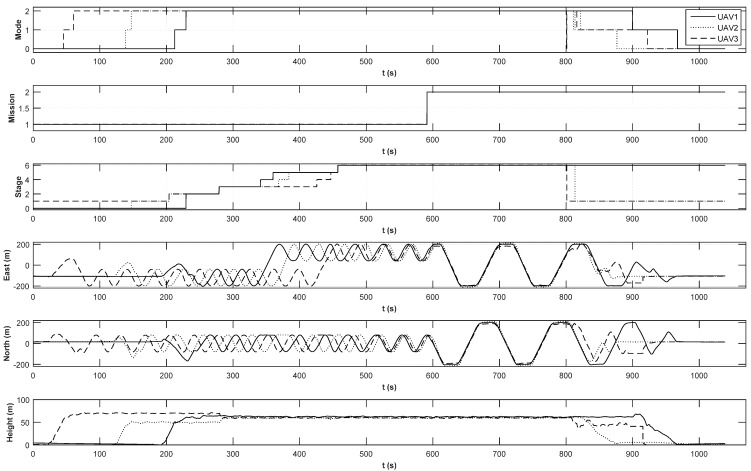
Position histories of integrated formation flight.

**Figure 17:**
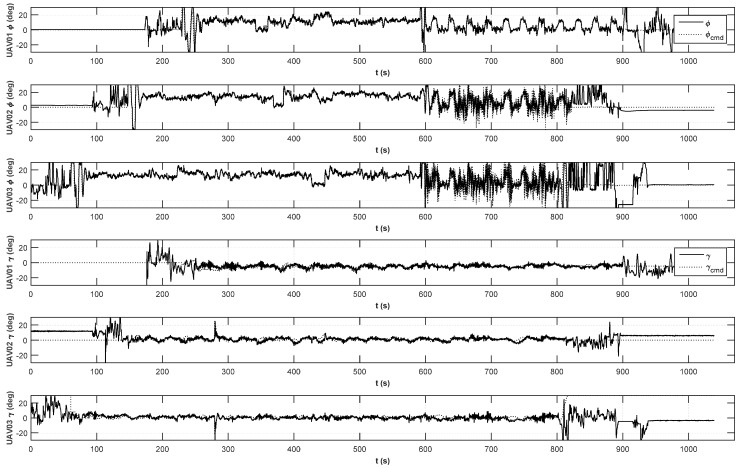
Lateral and longitudinal command histories of integrated formation flight.

**Figure 18:**
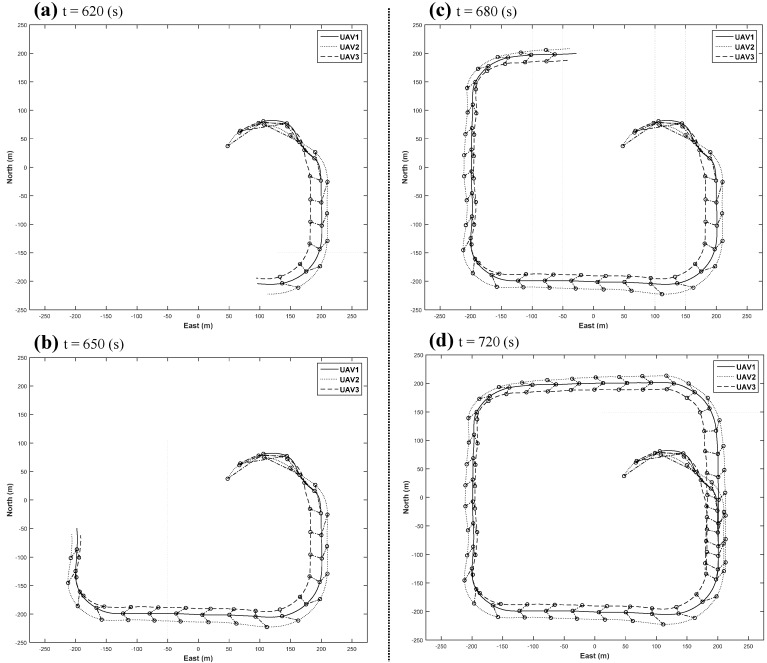
Detailed sequences of close triangular formation flight.

The position histories of the integrated formation flight are shown in Figure 16. The mode variable indicates a flight mode controlled by an RC controller, where Mode 0 is the manual flight mode, Mode 1 is the stabilized co-pilot flight mode, and Mode 2 is the automatic mission flight mode. Depending on the status of the UAVs, the stage of each UAV may be different. The circular formation flight starts at 230 s, and the triangular formation flight starts at 591 s. The lateral and longitudinal control histories are shown in Figure 17. As shown in Figure 17, the inner-loop controllers are found to perform well at following the guidance commands. A detailed triangular formation flight result is shown in Figure 18. As shown in Figure 18, UAV1 follows the rounded rectangular path, and the follower UAVs generate their own formation path based on the estimated path of UAV1. Due to the east wind effect, the formation paths of UAV2 and UAV3 are slightly shifted to the west; nevertheless, the triangular shape is maintained well during the formation flight.

**Figure 19:**
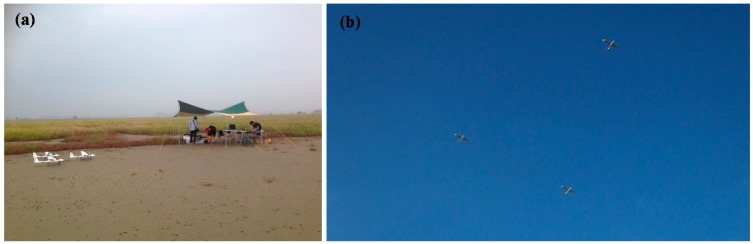
(**a**) Flight test setup; (**b**) Multiple UAVs in close triangular formation

Figure 19a shows the site of the flight test with the UAVs and the GCS, and Figure 19b shows the photo of the close formation flight using three UAVs in a triangular formation captured by a ground camera. The triangular formation is shown to be maintained well during the flight test [23].

## 6. Conclusions

The formation flight of three fixed-wing UAVs was performed based on circular formation flight guidance and a close formation flight algorithm. A multi-UAV system was developed, and the developed UAV dynamics were identified using a system identification scheme. A decentralized onboard sensor information sharing system for a miniature UAV system was developed using ZigBee modems, and the performance of the system was verified by analyzing the communication status between UAVs. Formation flight guidance laws of circular formation flight and close formation flight were proposed. The phase angle control scheme was used for circular formation flight, and the leader-follower guidance law was used for the close triangular formation flight during a formation movement. To verify the proposed algorithms for an entire formation flight, the integrated formation flight scenario was designed, and various formation flights were performed in sequence. The proposed guidance laws were first examined using a 6-DOF numerical simulation, and an actual flight test was conducted using the developed multi-UAV system.

## References

[1] Giulietti F., Pollini L., Innocenti M. Formation Flight Control: A Behavioral Approach. Proceedings of the AIAA Guidance, Navigation, and Control Conference.

[2] Price C.R. The Virtual UAV Leader. Proceedings of the AIAA Infotech.

[3] Li N.H.M., Liu H.H.T. Multiple UAVs Formation Flight Experiments Using Virtual Structure and Motion Synchronization. Proceedings of the AIAA Guidance, Navigation, and Control Conference.

[4] Teo R., Jang J.S., Tomlin C.J. Automated Multiple UAV Flight—The Stanford Dragon Fly UAV Program. Proceedings of the 43rd IEEE Conference on Decision and Control.

[5] Verma A., Wu C., Castelli V. UAV Formation Command and Control Management. Proceedings of the 2nd AIAA Unmanned Unlimited System, Technologies, and Operations.

[6] Schmitt L., Fichter W. (2014). Collision-Avoidance Framework for Small Fixed-Wing Unmanned Aerial Vehicles. J. Guid. Control. Dyn..

[7] Beard R.W., McLain T.W., Nelson D.B., Kingston D., Johanson D. (2006). Decentralized Cooperative Aerial Surveillance Using Fixed-Wing Miniature UAVs. IEEE Proc..

[8] Mahboubi Z., Kolter Z., Wang T., Bower G. Camera Based Localization for Autonomous UAV Formation Flight. Proceedings of the AIAA Infotech.

[9] Lee H.B., Moon S.W., Kim W.J., Kim H.J. (2013). Cooperative Surveillance and Boundary Tracking with Multiple Quadrotor UAVs. J. Inst. Control. Robot. Syst..

[10] Geoffrey B., Ted M., Michael L., Mark M., Joseph B. “Mini UAVs” for Atmospheric Measurements. Proceedings of the AIAA Infotech.

[11] Kim S., Oh H., Tsourdos A. Nonlinear Model Predictive Coordinated Standoff Tracking of Moving Ground Vehicle. Proceedings of the AIAA Guidance, Navigation, and Control Conference.

[12] Yamasaki T., Balakrishnan S.N., Takano H., Yamaguchi I. Coordinated Standoff Flights for Multiple UAVs via Second-Order Sliding Modes. Proceedings of the AIAA Guidance, Navigation, and Control Conference.

[13] Venkataramanan S., Dogan A. A Multi-UAV Simulation for Formation Reconfiguration. Proceedings of the AIAA Modeling and Simulation Technologies Conference.

[14] Gu Y., Seanor B., Campa G., Napolitano M.R., Rowe L., Gururajan S., Wan S. (2006). Design and Flight Testing Evaluation of Formation Control Laws. IEEE Trans. Control. Syst. Technol..

[15] Bayraktar S., Fainekos G.E., Pappas G.J. Experimental Cooperative Control of Fixed-Wing Unmanned Aerial Vehicles. Proceedings of the Conference on Decision and Control.

[16] Maza I., Kondak K., Bernard M., Ollero A. (2010). Multi-UAV Cooperation and Control for Load Transportation and Deployment. J. Intell. Robot. Syst..

[17] Park C., Kim H.J., Kim Y. Real-Time Leader-Follower UAV Formation Flight Based on Modified Nonlinear Guidance. Proceedings of the 29th Congress of the International Council of the Aeronautical Sciences.

[18] Oh G., Park C., Kim M., Park J., Kim Y. Small UAV System Identification in Time Domain. Proceedings of the Spring Conference of KSAS.

[19] Kim H.J., Kim M., Lim H., Park C., Yoon S., Lee D., Choi H., Oh G., Park J., Kim Y. (2013). Fully Autonomous Vision-Based Net-Recovery Landing System for a Fixed-Wing UAV. IEEE/ASME Trans. Mechatron..

[20] Park S., Deyst J., How J.P. (2007). Performance and Lyapunov Stability of a Nonlinear Path-Following Guidance Method. J. Guid. Control. Dyn..

[21] Kim D., Park S., Nam S., Suk J. A Modified Nonlinear Guidance Logic for a Leader-Follower Formation Flight of Two UAVs. Proceedings of the International Conference on Control, Automation Systems-SICE.

[22] Lee D., Lee J., Kim S., Suk J. (2014). Design of a Track Guidance Algorithm for Formation Flight of UAVs. J. Inst. Control. Robot. Syst..

[23] Formation Flight of Multiple UAV. https://youtu.be/6NVlgST9agQ.

